# Magnetically Induced Anisotropic Microstructures on Polyethylene Glycol Hydrogel Facilitate BMSC Alignment and Osteogenic Differentiation

**DOI:** 10.3390/gels10120814

**Published:** 2024-12-11

**Authors:** Hua Zhang, Yang Luo, Rong Xu, Xu Cao, Guanrong Li, Shang Chen

**Affiliations:** Research Institute of Smart Medicine and Biological Engineering, Health Science Center, Ningbo University, Ningbo 315211, China; xr1078000461@163.com (R.X.); 2311140169@nbu.edu.cn (X.C.); 13345966114@163.com (G.L.); ichenpi2004@gmail.com (S.C.)

**Keywords:** magnetic field induction, Fe_3_O_4_ micropatterns, oriented cytoskeleton, osteogenic differentiation

## Abstract

Many tissues exhibit structural anisotropy, which imparts orientation-specific properties and functions. However, recapitulating the cellular patterns found in anisotropic tissues presents a remarkable challenge, particularly when using soft and wet hydrogels. Herein, we develop self-assembled anisotropic magnetic Fe_3_O_4_ micropatterns on polyethylene glycol hydrogels utilizing dipole–dipole interactions. Under the influence of a static magnetic field, Fe_3_O_4_ nanoparticles align into highly ordered structures with a height of 400–600 nm and a width of 8–10 μm. Furthermore, our layer-by-layer assembly technique enables the creation of oriented micropatterns with varying densities and heights, which can be further manipulated to form three-dimensional structures by adjusting the angle of the magnetic field. These anisotropic magnetic Fe_3_O_4_ micropatterns can be applied to various substrates, including treated glass slides, standard glass slides, silicon wafers, and polydimethylsiloxane. The patterned Fe_3_O_4_ scaffolds, modified with gold coating, effectively enhance cellular adhesion, orientation, and osteogenic differentiation of bone marrow-derived stem cells, which is crucial for effective tissue repair. Overall, this study presents an efficient strategy for constructing anisotropic Fe_3_O_4_ micropattern hydrogels, providing a bioactive platform that significantly enhances cellular functions.

## 1. Introduction

Biological tissues, such as the periosteum, cartilage, muscle, blood vessel, cornea, esophagus, etc., exhibit multi-level, oriented microstructures ranging from the nanometer to the micron scale. These structures endow tissues with exceptional adaptive biological functionality. For example, the esophageal tissue comprises both inner link and outer longitudinal muscle layers, characterized by an organized collagen fiber extracellular matrix (ECM) [1]. This ECM guides endothelial and smooth muscle cells, facilitating the formation of distinct inner and outer growth structures, which endow the esophagus with remarkable contractile and peristaltic properties. However, diseases, such as tumors or trauma, lead to tissue and organ defects, severely impairing their functional performance. Therefore, the in vitro fabrication of biomimetic tissues that replicate the anisotropic structures found in vivo is of paramount importance for repairing these defects and restoring normal function [2,3,4]. A plethora of studies have dedicated efforts toward engineering cells into specific patterns and morphologies. Photolithographic techniques, providing substrates with striped and pitted geometries, facilitate the oriented growth and differentiation of cells [5]. Techniques such as electrospinning and electrical stimulation offer simple approaches to induce cell alignment on two-dimensional (2D) planes [6,7,8,9]. Furthermore, three-dimensional (3D) printing technology empowers cells with the capacity for self-organization within complex spatial structures [10,11,12,13]. The advancement of these technologies propels the elaboration of cells into sophisticated engineered tissues. The ECM of various tissues has been extensively studied for inducing orderly cell growth and promoting tissue repair. Nonetheless, the ECM contains numerous complex physical and molecular signals, making it challenging to precisely control cell morphogenesis and function through oriented features [14]. Therefore, designing and constructing an in vitro microenvironment that mimics these highly oriented nano- and microstructures not only is beneficial for studying cellular perception and responses to micro- or nano-scale features but also holds significant importance for efficient tissue repair and regeneration.

Numerous strategies have been developed to construct an artificial matrix with orientation structures by using various methods, such as self-assembly [15,16], strain alignment [17,18], directional freeze–thawing [19,20], electric field alignment [21,22], and magnetic field alignment [23,24]. Among them, magnetic field orientation has the unique advantages of noncontact, excellent responsiveness, and high biocompatibility. It can flexibly manipulate zero-dimensional, one-dimensional, and two-dimensional magnetic nanoparticles for long-range ordered assembly [25,26,27]. For example, Pardo et al. successfully engineered a biomimetic hydrogel construct with controlled anisotropic architectures by combining magnetically and matrix-assisted 3D bioprinting strategies [28]. This anisotropic hydrogel effectively directed the fate of the encapsulated human adipose-derived stem cells toward a tenogenic phenotype. Recently, we developed an iron oxide (Fe_3_O_4_) filament-embedded gelatin–silk fibroin composite hydrogel by using magnetic induction and bioprinting [29]. The bioprinted muscle-like matrices promoted the self-organization of smooth muscle cells (SMCs) and the directional differentiation of bone marrow mesenchymal stem cells (BMSCs) into SMCs. These studies demonstrated that magnetic nanoparticles and their self-assembled microfibers can form highly anisotropic microstructures within hydrogel scaffolds, guiding cell alignment and biomimetic tissue formation. However, the fabrication of anisotropic tissue structures using 3D anisotropic hydrogels may encounter issues such as low cell viability, slow cell spreading, and low orientation.

In the context of various wound repairs—such as the regeneration of the esophageal mucosa, the healing of superficial skin injuries, and the repair of periosteal defects—2D anisotropic scaffolds have shown greater efficiency in generating anisotropic cell structures. Research indicates that two-dimensional surfaces facilitate more controlled cellular interactions and alignment due to their simpler geometry and more predictable mechanical properties [30,31,32]. This allows for more effective cell migration and proliferation, which are essential for successful tissue regeneration. Furthermore, 2D anisotropic scaffolds serve as a valuable platform for studying the interactions between cells and anisotropic matrices. These surfaces enable researchers to investigate how cellular behavior is influenced by matrix anisotropy, allowing for the optimization of scaffold design to promote desired cellular responses. The ability to manipulate surface properties, such as topography and chemical cues, enhances our understanding of cell–matrix interactions, which is critical for developing effective tissue engineering strategies. However, the formation of highly oriented magnetic assemblies on the surface of hydrogels remains a significant challenge due to their inherent soft and wet structure.

In this study, we develop anisotropic magnetic Fe_3_O_4_ microstructures on polyethylene glycol hydrogel through the utilization of dipole–dipole interactions. When subjected to a static magnetic field, Fe_3_O_4_ nanoparticles undergo rapid self-assembly, aligning themselves with the direction of the magnetic field. By carefully controlling parameters, such as the concentration of Fe_3_O_4_, the number of assembly layers, and the direction of assembly, we can fabricate micropatterns that vary in spacing, height, and orientation. Furthermore, when BMSCs are seeded onto these micropatterned substrates, they demonstrate anisotropic adhesion and enhanced osteogenic differentiation. The results highlight the potential of these structures for application in tissue engineering.

## 2. Results and Discussion

### 2.1. Fabrication of Anisotropic Magnetic Architectures

**Figure 1:**
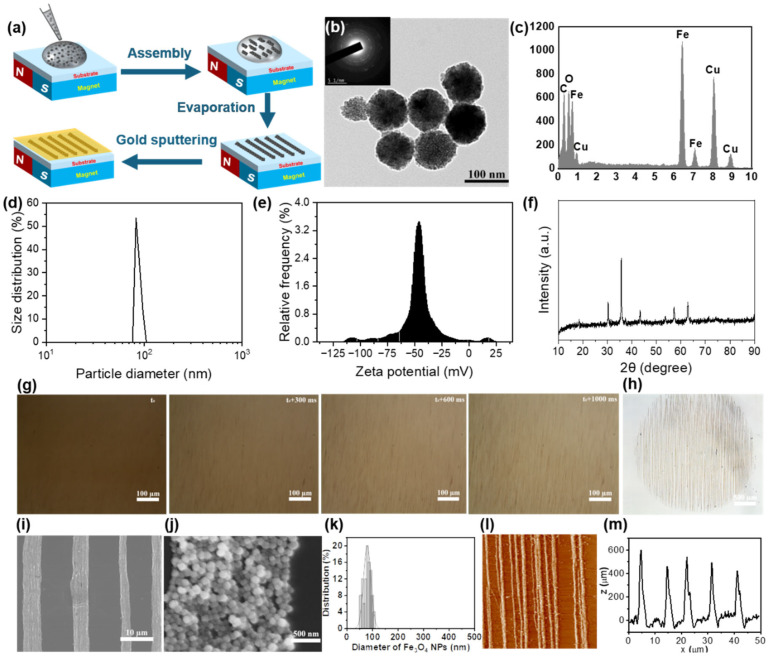
Fabrication of anisotropic micropatterns using a magnetostatic field. (**a**) Schematic illustration of the preparation process for creating oriented micropatterns. (**b**) TEM image of Fe_3_O_4_ NPs. (**c**) EDS elemental mapping demonstrating the crystalline structure of Fe_3_O_4_ NPs, with oxygen and iron as the predominant elements. (**d**,**e**) The size distribution (**d**) and zeta potential (**e**) of the Fe_3_O_4_ NPs characterized by using the zetasizer nano analyzer. (**f**) XRD pattern of the magnetic Fe_3_O_4_ NPs. (**g**) Assembly process of Fe_3_O_4_ NPs observed using an optical microscope. (**h**) Optical microscopy images of Fe_3_O_4_ micropatterns. (**i**,**j**) SEM morphology images showing the morphology of Fe_3_O_4_ micropatterns. (**k**) Statistical analysis of the diameter distribution of Fe_3_O_4_ NPs using SEM. (**l**) AFM images and (**m**) quantitative analysis on the height and width of the oriented Fe_3_O_4_ micropatterns.

We fabricated a series of linear and multilayer cross patterns of Fe_3_O_4_ on PEG hydrogels using a simple method based on magnetostatic field induction and surface gold spraying. Specifically, when a drop of a magnetic Fe_3_O_4_ NP dispersion is deposited onto a PEG hydrogel scaffold, the Fe_3_O_4_ NPs rapidly self-assemble into anisotropic magnetic assemblies under the induction of a static magnetic field (Figure 1a). Following the evaporation of the solvent, the PEG hydrogel scaffold underwent gold sputter coating, enhancing its cell adhesion properties. These Fe_3_O_4_ NPs exhibited regular spherical morphology with an average particle size of 80 nm, accompanied by characteristic crystalline phase diffraction rings, as confirmed by transmission electron microscopy (TEM) observation (Figure 1b). Energy-dispersive X-ray spectroscopy (EDS) elemental mapping revealed a crystalline structure of the Fe_3_O_4_ NPs, predominantly comprising oxygen and iron, confirming the successful synthesis of the Fe_3_O_4_ NPs (Figure 1c). The size distribution of Fe_3_O_4_ NPs was found to be approximately 80 nm, as confirmed by dynamic light scattering (Figure 1d). This finding aligns with observations made using TEM, reinforcing the reliability of our measurements. Additionally, the measured zeta potential values ranged from approximately −110 mV to +25 mV, indicating a stable suspension of the nanoparticles in deionized water (Figure 1e). Furthermore, the X-ray diffraction (XRD) patterns displayed a series of spinel-type diffraction peaks, which can be indexed to the face-centered cubic structure of Fe_3_O_4_ NPs (Figure 1f).

The assembly process of Fe_3_O_4_ NPs under the influence of the magnetostatic field was monitored in situ using an optical microscope equipped with a high-resolution camera (Video S1). When 10 μL droplets of Fe_3_O_4_ aqueous solution (0.25 mg/mL) were deposited on the PEG hydrogel substrate, they formed a hemispherical liquid pattern. The application of the static magnetic field rapidly polarized the spherical Fe_3_O_4_ NPs, leading to the assembly of nearby particles into clearly visible parallel clusters under the microscope. These clusters then grew into compact structures within just one second, as illustrated in Figure 1d. Remarkably, the entire assembly process occurred within just one second. After the evaporation of water, the highly ordered self-assembled structures of Fe_3_O_4_ became evident (Figure 1e), highlighting the efficiency of the assembly process. SEM and atomic force microscopy (AFM) were employed to characterize the filaments of Fe_3_O_4_ nanoparticles fabricated through magnetic dipole-dipole interactions. The SEM analysis showed that the Fe_3_O_4_ NPs aggregate closely into oriented microfilaments, exhibiting a homogeneous size of approximately 80 nm (Figure 1f–h). AFM images further provided high-resolution three-dimensional surface morphology maps, clearly illustrating the microstructural characteristics of the Fe_3_O_4_ micropatterns (Figure 1i). The surface profile revealed that the height of these filaments ranged from 400 to 600 nm, with distances between adjacent lines measuring 8 to 10 μm (Figure 1j). However, it was observed that some Fe_3_O_4_ clusters remained between adjacent parallel microfilaments, resulting in widths and intervals that were not uniform. This irregularity may be attributed to the strong magnetostatic field, which attracted the Fe_3_O_4_ nanoparticles downward during the drop application, preventing the clusters from relocating to their precise positions in time.

### 2.2. Optimization of Highly Oriented Architectures

**Figure 2:**
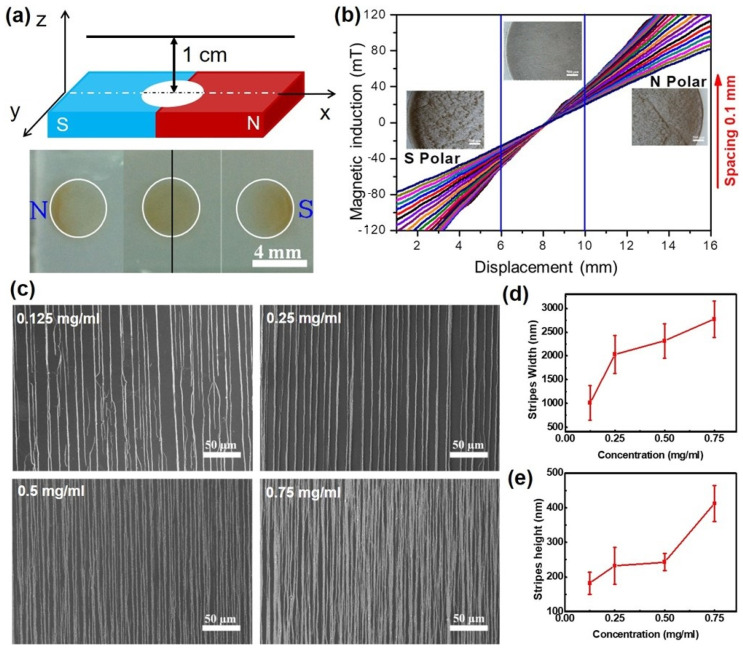
Parameter optimization for the fabrication of anisotropic micropatterns. (**a**) Distribution of Fe_3_O_4_ droplets at varying positions on the magnet. (**b**) The uniform micropatterns stabilized at the magnet positions within a 4 mm diameter. (**c**) SEM images of magnetically induced anisotropic Fe_3_O_4_ micropatterns at different concentrations. (**d**,**e**) Statistical analysis of the width (**d**) and height (**e**) of the anisotropic Fe_3_O_4_ micropatterns.

A crucial aspect of the assembly technique involves the precise positioning of droplets on a static magnet. This positioning is essential to prevent superparamagnetic nanoparticles from being attracted to the local magnetic field gradient maximum (Figure 2a). The uniform stripe structures could be stabilized at the positions on both sides of the interface region, which has a diameter of 4 mm, as well as at positions perpendicular to this region (Figure 2b). When an aqueous solution of Fe_3_O_4_ nanoparticles is dripped onto the magnet, regardless of whether it is directed slightly toward the south (S) or north (N) pole, the mutual forces between the magnetic dipoles and the magnetic field attract the nanoparticles to the local magnetic field gradient maximum. This attraction led to irregular assembly and non-uniform distributions. Consequently, optimizing the droplet positioning and controlling the magnetic field distribution are critical for achieving uniform assembly.

The concentration of Fe_3_O_4_ NPs is another crucial control parameter that influences magnetic dipole–dipole interactions, which are essential for fabricating integrated and regular patterns. To investigate the effect of Fe_3_O_4_ NP concentration on pattern formation, we selected a dispersion liquid with concentrations ranging from 0.125 to 0.75 mg/mL (*w*/*v*) (Figure 2c). At a concentration of 0.125 mg/mL, the formed Fe_3_O_4_ lines had widths and heights of approximately 500–1500 nm and 150–200 nm, respectively; however, some breakpoints were observed in these lines, which could significantly impact the morphology and performance of the patterned devices. In contrast, when we increased the concentration to 0.75 mg/mL, the Fe_3_O_4_ lines became thicker and denser (Figure 2d,e). This indicates that as the concentration increases, there is a tendency for the particles to coalesce into a more uniform film. To achieve optimal integrity and regularity in the patterns, we selected a concentration of 0.25 mg/mL for depositing linear and hierarchical structures on PEG and other substrates in these experiments.

**Figure 3:**
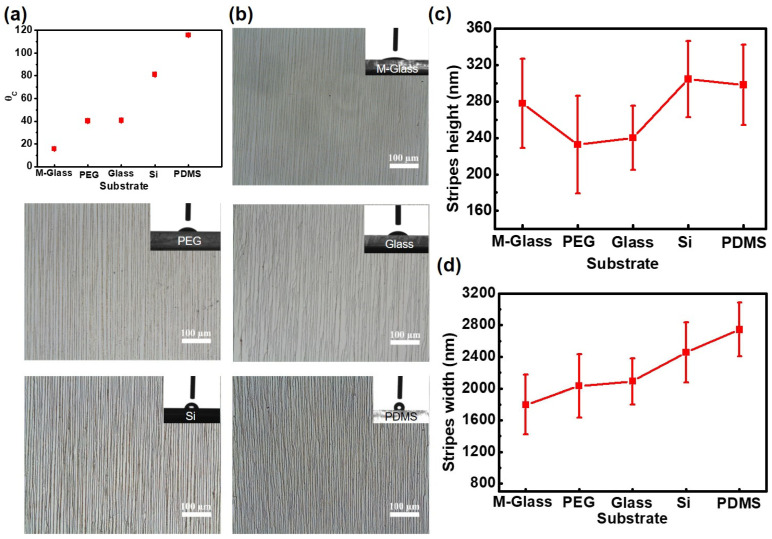
The morphology of Fe_3_O_4_ micropatterns on substrates with varying wettability. (**a**) Contact angles measured on five different supporting substrates. (**b**) Anisotropic Fe_3_O_4_ micropatterns formed on substrates with different wettability. (**c**,**d**) The height (**c**) and width (**d**) of Fe_3_O_4_ micropatterns on these various substrates.

The feature size of printed dots or lines is closely related to the contact angle of the liquid droplets on a substrate, which primarily depends on the substrate’s wettability. To enhance the control and feasibility of this patterning approach, we investigated how substrate wettability affects the deposited patterns. We selected five types of supporting substrates with varying wettability: a glass slide treated with phinas solution (M-Glass), polyethylene glycol (PEG), a standard glass slide, a silicon wafer, and polydimethylsiloxane (PDMS). The corresponding contact angles for these substrates were measured at 20°, 40°, 45°, 80°, and 120° (Figure 3a). The Fe_3_O_4_ NPs formed distinct deposition patterns on all five substrates when using this method (Figure 3b). Notable, the wettability of the substrate significantly influenced the spreading behavior of the Fe_3_O_4_ droplets, which in turn affected the height, width, and arrangement density of the deposited patterns. Notably, substrates with larger contact angles tended to produce wider Fe_3_O_4_ stripes but did not necessarily lead to increased heights of the deposits (Figure 3c,d).

### 2.3. Fabrication of Multilayer Cross Patterns

**Figure 4:**
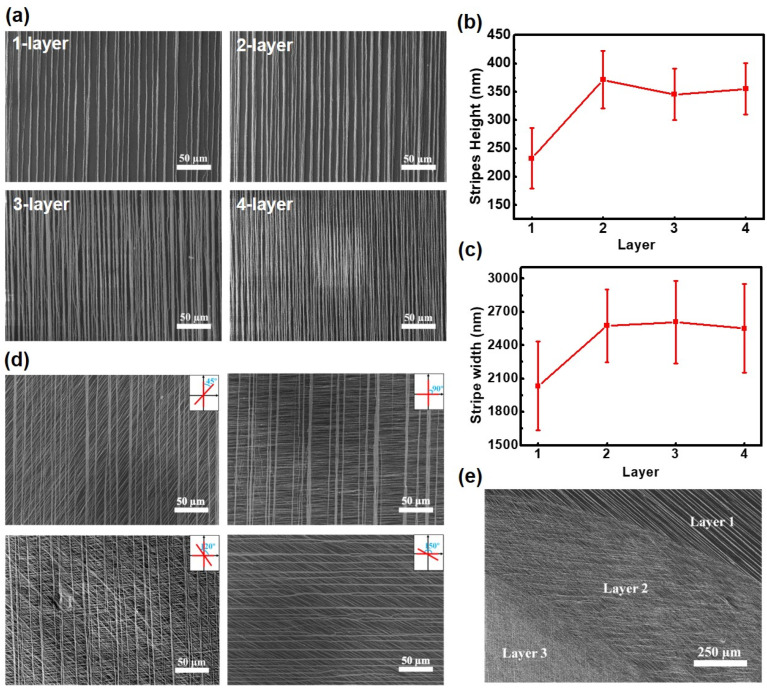
Fabrication of multilayer cross patterns. (**a**) SEM images of multilayer parallel structures, consisting of one to four layers, on PEG surfaces. (**b**,**c**) The height (**b**) and width (**c**) of multilayer Fe_3_O_4_ micropatterns. (**d**) SEM images of Fe_3_O_4_ micropatterns created at angles of 45°, 90° 120° and 150° direction. (**e**) SEM image of three-layer Fe_3_O_4_ cross structures.

By assembling Fe_3_O_4_ patterns sequentially, it is possible to form hierarchical structures using this magnetic assembly technique. We present multilayer structures that consist of both oriented and cross architectures, which are assembled on PEG surfaces through a layer-by-layer deposition method. As the number of assembled layers increases, the stripes in Figure 4a become denser. Specifically, the heights and widths of the parallel structures increase from 232.6 nm and 2033.7 nm to 355.5 nm and 2552.2 nm, respectively, as the number of layers increases from one to two. However, the dimensions do not continue to increase with additional layers since the Fe_3_O_4_ NPs not only deposit on the previous stripes but also assemble in the gaps between them (Figure 4b,c).

In the cross-layer structures, the stripes deposited in subsequent steps were oriented at specific angles to the magnetic field created by the magnet, which was set at 45°, 90°, 120°, and 150° (Figure 4d). Notably, in these cross two-layer and three-layer structures, there was mutual noninterference between each Fe_3_O_4_ layer in the presence of the magnetic field (Figure 4e and Video S2). This lack of interference was primarily due to the magnetic nanoparticles deposited in the first step becoming trapped by the substrate, preventing them from moving in response to the perpendicular and arbitrary field applied in the second and subsequent steps. Therefore, this patterning approach allows for the creation of diverse patterns on various substrates.

### 2.4. Cellular Alignment and Osteogenic Differentiation on Patterned Hydrogels

**Figure 5:**
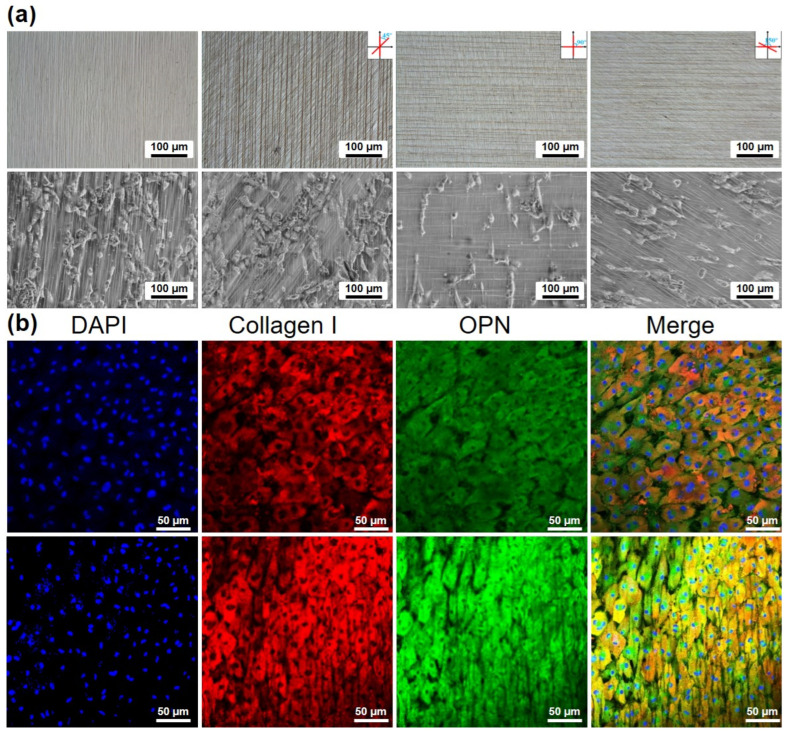
Cellular orientation and differentiation on micropatterned substrates. (**a**) Cellular orientation on anisotropic Fe_3_O_4_ micropatterns and multilayer patterns. (**b**) Cellular orientation on single-layer linear patterns formed by the assembly of varying concentrations of Fe_3_O_4_ NPs. (**b**) Representative fluorescent images showing nuclear (blue), Collagen I (red), and OPN (green) immunostaining of BMSCs differentiated on the micropatterned Fe_3_O_4_ hydrogels.

The micropatterned substrates provide discrete topographical cues that guide the planar alignment of cells. We prepared single-layer oriented microstripes and double-layer microstripes stacked at angles of 45°, 90°, and 150° and seeded rabbit bone marrow-derived mesenchymal stem cells (BMSCs) onto their surfaces. As shown in Figure 5a, the cells exhibited an organized adhesive morphology on the micropatterned hydrogel scaffold, primarily sensing the upper pattern structure. These results indicate that the patterned Fe_3_O_4_ scaffolds can effectively regulate cell morphology on a lateral scale while also demonstrating the perceptual range of cells on a vertical scale. Numerous studies have demonstrated that physical cues in the microenvironment can control the fate of stem cells, and such specific settings can induce osteogenic differentiation of stem cells [33]. Our research investigated the osteogenic differentiation of stem cells by altering the substrate for cell attachment, which is consistent with the literature reports [34,35,36]. Furthermore, we investigated the effects of oriented Fe_3_O_4_ scaffolds on the osteogenic differentiation of BMSCs, using isotropic Fe_3_O_4_ scaffolds as a control. As illustrated in Figure 5b, under osteogenic differentiation induction, BMSCs expressed Collagen I and osteopontin (OPN) proteins on both isotropic and anisotropic Fe_3_O_4_ scaffolds. Notably, the expression levels of these proteins in cells on the anisotropic Fe_3_O_4_ scaffolds were significantly higher than those observed on the isotropic scaffolds. These results suggest that the oriented Fe_3_O_4_ scaffolds promote the osteogenic differentiation of BMSCs more effectively than their isotropic counterparts. Consequently, the anisotropic Fe_3_O_4_ micropatterns provide a bioactive platform to enhance cellular functions. In future studies, by optimizing the topographical features of these scaffolds, we can create an environment that not only supports cell alignment and proliferation but also facilitates specific differentiation pathways. This capability is crucial for the development of advanced tissue engineering applications, where precise control over cell behavior is essential for the successful integration and regeneration of tissues.

## 3. Conclusions

In conclusion, we have developed an effective method for constructing linear and hierarchical patterns based on dipole–dipole interactions. We successfully fabricated a series of linear and multilayered Fe_3_O_4_ micropatterns on both hydrophilic and hydrophobic substrates. These micropatterned substrates facilitated the oriented alignment of BMSCs and significantly enhanced their osteogenic differentiation, which is crucial for advancing targeted bone repair therapies. Moreover, this system can be utilized to explore the directed differentiation of stem cells into various cell types, establishing specific interfacial requirements for material design and processing. This capability is particularly beneficial for the targeted repair of damaged tissues and organs using cell-laden scaffolds. Furthermore, the ability to manipulate cell alignment and differentiation through the design of anisotropic scaffolds opens new possibilities for creating anisotropic tissues in vitro. This approach could lead to the development of biomimetic tissues and organs that closely resemble physiological structures. Overall, our findings present exciting new avenues for innovative applications in regenerative medicine and tissue engineering, which will be a primary focus of our future research endeavors.

## 4. Materials and Methods

### 4.1. Materials

All materials were obtained from the suppliers as follows and were used as received. Iron (II) sulfates, iron (III) chlorides, oligo (ethylene glycol) diacrylate (oEGDA) (MW = 547), and 2-hydroxy-40-(2-hydroxyethoxy)-2-methylpropiophenone were purchased from Sigma-Aldrich. The components of Dimethylsiloxane (DMS) (Sylgard 184, A (base: dimethylsiloxane): B (curing agent: methylhydrogensiloxane) = 1:10) were obtained from Dow Company.

### 4.2. Preparation of Fe_3_O_4_ Nanoparticles

In order to achieve particles of different sizes, Fe_3_O_4_ NPs were prepared by using the solvothermal method and chemical co-precipitation method according to the literature. In a typical synthesis of Fe_3_O_4_ NPs by the solvothermal method, Fe_3_O_4_ NPs were synthesized by hydrolysis of iron (III) chloride hexahydrate at 200 °C. Briefly, 0.318 g of trisodium citrate dehydrate was added to 20 mL of ethylene glycol (EG) by stirring for 1 h to form stock. After the trisodium citrate dehydrate was completely dissolved, 1.8 g of sodium acetate anhydrous and 1.1 mL of deionized water were added to stock A, which was stirred for an additional hour. Meanwhile, 0.819 g of iron (III) chloride hexahydrate was dissolved in 10 mL of EG to form stock B. Then, stock B was quickly injected into stock A by stirring for 2 h, resulting in a black solution. Finally, the mixture was transferred to a reaction vessel and maintained at 200 °C for 10 h.

### 4.3. Preparation of Different Wettability Substrates

Different materials were used to fabricate substrates with different wettability. The PEG gel was prepared by polymerizing oligo (ethylene glycol) diacrylate (oEGDA) (M_W_ = 547) via ultraviolet irradiation (365 nm, 1 min) by using 2-hydroxy-4′-(2-hydroxyethoxy)-2-methylpropiophenone as the initiator. The glass slides and wafers were rinsed clean with deionized water and below-dried with N_2_. The more hydrophilic glass slides were further cleaned in a mixture solution of H_2_O_2_/H_2_SO_4_ (1:3, *v*/*v*) (“piranha solution”) at 70 °C for 1 h, washed thoroughly with deionized water, and blow-dried with N_2_. The soft polydimethylsiloxane (PDMS) (Sylgard 184, A:B = 1:10, Dow Company, Midland, MI, USA) substrate was fabricated by mixing components A and B at 90 °C for 4 h.

### 4.4. Fabrication of Anisotropic Fe_3_O_4_ Micropatterns

The magnetostatic field used for the Fe_3_O_4_ NPs assembly was created with an 8 cm × 4 cm × 2 cm magnet. The substrates, including PEG, glass, wafer, and PDMS, were positioned in the center of the magnet. And then, a drop of Fe_3_O_4_ solution (10 μL) was dripped on the substrates. Finally, the water was evaporated at room temperature, and it took about 3 to 5 min. The multilayer cross patterns were obtained by layer-by-layer assembly processes of the linear Fe_3_O_4_ patterns.

### 4.5. Micropattern Characterization

The structure and morphology of the Fe_3_O_4_ self-assembly patterns produced on different substrates were characterized by optical microscopy (Olympus BX51, Olympus Life Science, Waltham, MA, USA) in the reflection mode, scanning electronic microscopy (SEM, Hitachi S-4800, Hitachi, Tokyo, Japan), and atomic force microscopy (CSPM 5500, Benyuan Nano Instruments Co., Ltd., Guangzhou, China) in the tapping mode. The spring constant of scanning probes is 40 N m^−^^1^. The X-ray diffraction (XRD) measurements were conducted using a Bruker D2 X-ray diffractometer (Bruker Corporation, Bremen, Germany) to analyze the crystallographic structure of the samples. Additionally, the zeta potentials of the particles were measured using electrophoretic light scattering with a Zetasizer Nano-ZS (Malvern, Worcestershire, UK), which also provided measurements of the particle diameters. The size and distribution of Fe_3_O_4_ NPs were characterized by an FEI Tecnai F20 transmission electron microscope (FEI, Hillsboro, OR, USA) operated at 200 kV, and the samples were prepared by dropping the solution onto a carbon-coated copper grid. Static contact angles were measured on a Dataphysics OCA20 contact-angle system (Dataphysics, Stuttgart, Germany) at ambient temperature. The average contact angle was obtained by measuring more than four different positions of the same substrate. The contact angles and contact diameters during the evolution process were recorded by the Dataphysics OCA20 contact-angle system equipped with a bright-field optical microscope and CCD video camera.

### 4.6. Cellular Orientation on Micropatterned Substrates

BMSCs were extracted from the bone marrow of rabbits by the adherent method and cultured in a Roswell Park Memorial Institute 1640 medium (RPMI1640, VivaCell Biosciences, Denzlingen, Germany) supplemented with 10% fetal bovine serum (FBS, VivaCell Biosciences) and 1% penicillin/streptomycin (NCM biotech, Suzhou, China). BMSCs were inoculated on micropatterned substrates and cultured for 48 h. The cells were observed by using an optical microscope (NeXcope, Ningbo, China).

### 4.7. In Vitro Osteogenic Differentiation

To evaluate the osteogenic differentiation potential of bone marrow stem cells (BMSCs) in vitro, the cells were first seeded onto micropatterned hydrogels and then cultured in an osteogenic differentiation induction medium. After 21 days of culture, immunofluorescence staining was performed to assess the expression of osteogenic markers. Following three washes with phosphate-buffered saline (PBS), the cells were fixed and permeabilized for one hour. To block non-specific binding, the cells were treated with 3% bovine serum albumin (BSA, Beyotime, Haimen, China) for six hours. Subsequently, the cells were incubated overnight at 4 °C with primary antibodies against osteopontin (OPN) at a dilution of 1:500 (Proteintech, Rosemont, IL, USA, 22952-1-AP) and Collagen I at a dilution of 1:500 (Proteintech, USA, 67288-1-Ig). The corresponding secondary antibody was stained at room temperature for 6 h. After three PBS washes, the cell morphology was observed and imaged using a confocal laser scanning microscope (CLSM, STELLARIS 5, Leica, Wetzlar, Germany).

## Data Availability

All data that support the findings of this study are included within the article.

## Supplementary Materials

The following supporting information can be downloaded at: https://www.mdpi.com/article/10.3390/gels10120814/s1, Video S1: Microscopic in situ observation of the formation of anisotropic Fe_3_O_4_ micropatterns on the surface of PEG hydrogel. Video S2: Microscopic in situ observation of the formation of bilayer Fe_3_O_4_ micropatterns on the surface of PEG hydrogel.
